# Ionic basis of a mechanotransduction current in adult rat dorsal root ganglion neurons

**DOI:** 10.1186/1744-8069-2-28

**Published:** 2006-08-21

**Authors:** Gordon C McCarter, Jon D Levine

**Affiliations:** 1 Department of Oral and Maxillofacial Surgery, Division of Neurosciences, University of California at San Francisco, San Francisco, CA 94143-0440, USA; 2 College of Pharmacy, Touro University – California, 1310 Johnson Lane, Mare Island, Vallejo, CA 94592-1118, USA

## Abstract

Sensory mechanical transduction – necessary for hearing, proprioception, and the senses of touch and pain – remains poorly understood. In somatosensation, even the basic properties of the mechanically sensitive excitatory ionic currents that are assumed to mediate mechanical transduction are largely undescribed. We have recorded, from the soma of rat dorsal root ganglion (DRG) neurons *in vitro*, whole-cell ionic currents induced by the impact of a piezo-electrically driven glass probe. This transient mechanically activated current was observed in virtually all DRG neurons tested. In ion substitution experiments the current could be carried nonselectively by most cations, including divalent and organic cations, but not by chloride or sulfate ions. In addition, the mechanically activated current carried by monovalent cations was consistently blocked by millimolar concentrations of external calcium or magnesium. Based on these results, the transient mechanical transduction current observed in somatosensory neurons *in vitro *is mediated by large-pore mechanically gated channels nonselective for cations but impermeable to anions.

## Background

All animals employ mechanical sensation to interpret their external and internal environments. The transduction of thermal and chemical stimuli by sensory neurons has been well described physiologically, and molecules mediating transduction for several of these signals have been identified [1,2]. In contrast, mechanical transduction is poorly understood and the molecules by which mechanical energy activates sensory neurons remain largely unidentified and their actions have not been well characterized.

One reason that somatosensory mechanical transduction is poorly understood is the difficulty in directly observing it, as the nerve terminals where it occurs *in vivo *are sparsely distributed and sub-micron in diameter, making them inaccessibly small for electrical or biochemical examination. Single-channel studies have described stretch-activated cation channels from DRG neurons *in vitro *[3], but it has been difficult to correlate single-channel currents elicited by tension in isolated patches of membrane to whole-cell currents evoked by forces acting on larger structures. Corroborating whole-cell data is thus required to establish the relationship of the single-channel data to macroscopic events. Membrane depolarization and calcium influx can be triggered by osmotic forces on DRG neurons *in vitro *[4,5], but the underlying currents have not been described. Also, it is not clear how such currents would relate to those that are elicited on a shorter time scale from direct contact and membrane deformation by a foreign object, a stimulus that may be more physiologic in relation to the sense of touch and acute pain.

We have previously described an *in vitro *system in which rat sensory neuron somata can be mechanically stimulated during whole-cell voltage-clamp recording [6]. We observed a fast nonselective cation current, fully activating within 1–10 ms, that displayed graded responses to the impact of a fluid jet or a piezo-electrically driven glass probe. Few reports have been published describing currents in response to direct contact with the DRG neuronal soma [7-9], while similar fast currents in response to transient increases in intracellular pressure have been briefly described [3,10]. Thus, many questions remain regarding the basic properties of fast whole-cell mechanotransduction currents and their relation to mechanosensation observed *in vivo*.

One of the most fundamental properties of a current – providing a signature to aid in the molecular identification of the underlying channel – is its ability to be carried by different ionic species. A detailed description of the ionic basis of somatosensory mechanotransduction has not yet been performed. We have therefore recorded mechanosensitive whole-cell currents in rat dorsal root ganglion (DRG) neurons *in vitro *in order to determine the relative permeability of the mechanosensitive channels to a variety of ions.

## Results

### Mechanically activated current in DRG neurons

**Figure 1:**
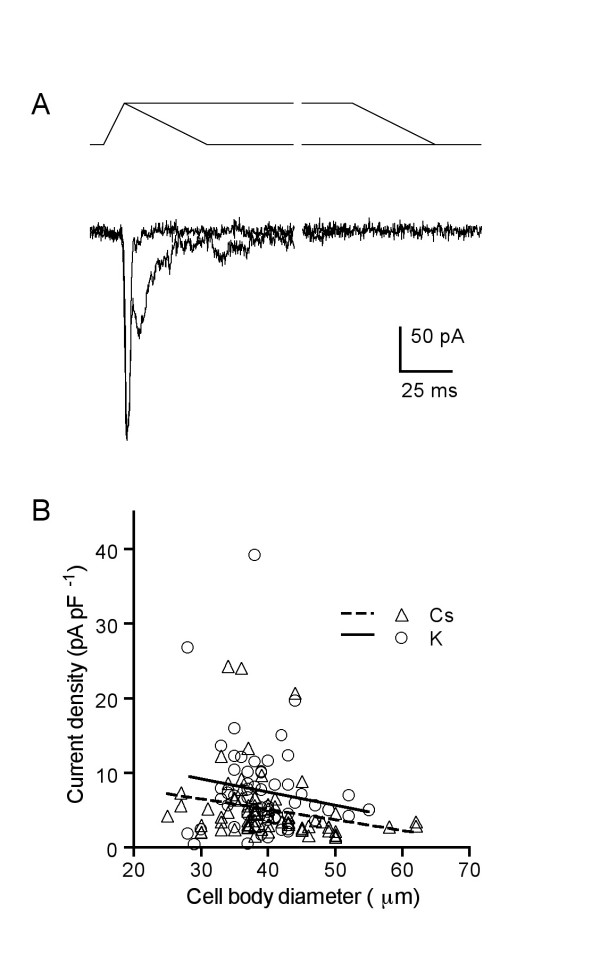
**Mechanical stimulus elicits fast transient currents in most DRG neurons**. (A) Representative traces of currents in a typical neuron (36-μm diameter) in response to a transient and a sustained mechanical stimulus at -70 mV. The travel of the probe is shown at top, returning to start position immediately for one stimulus and remaining in contact with the neuron for 200 ms for the other. The probe travelled 33 μm; therefore its velocity was 3.3 mm/s. Both responses displayed an identical initial fast component but when the neuron was transiently stimulated the current inactivated within 4 ms, while the sustained stimulus elicited additional later components – possibly induced by residual vibration of the probe – that were completely inactivated within ~75 ms. The electrode contained the potassium-based electrode solution. (B) Largest current amplitude recorded from a series of DRG neurons plotted against the cell soma diameter. Each point represents the largest current evoked from a single neuron at -70 mV using electrodes filled with either a potassium- (○) or cesium-based (△) electrode solution. All cells from 35 preparations (n = 133) in which a stable recording could be maintained through the initial mechanical stimulation are included. Setting the threshold for a response at 1 pA/pF, 98% of the DRG neurons tested expressed the MA current. Solid and dashed lines represent a linear regression of the data for cells recorded using electrodes filled with potassium- and cesium-based electrode solution, respectively.

In our mechanical stimulation protocol the distance and hence velocity at which the probe moved in its 10-ms approach was calibrated for each cell to elicit near-maximal responses without disrupting the patch-clamp recording. Virtually all DRG neurons tested responded to this stimulus with an inward, rapidly activating and inactivating current. This contrasts with post-ganglionic sympathetic neurons in which mechanosensitive currents were never observed using the same stimulation protocol [6]. Figure 1A shows traces from a typical neuron, in which the mechanically activated (MA) current was repeatedly elicited by transient and sustained stimuli of the same approach velocity. When the probe was immediately withdrawn after reaching its maximal travel distance and impacting the neuron, the current became half-inactivated within 2 ms. When the probe was not immediately withdrawn and remained in contact with the neuron for 200 ms, continuing to deform the membrane, the current exhibited an initial fast peak identical to that evoked by the immediately withdrawn stimulus but also comprised more prolonged components with at least two sub-peaks (Fig. 1A). However, the current evoked by the sustained stimulus still inactivated completely during contact with the probe. The roughly oscillatory pattern of the residual current was superimposable from trial to trial and suggests that vibration of the probe at the end of forward motion may continue to stimulate the neuron during this period. In 18 neurons tested with the prolonged stimulus (mean diameter 40 ± 2 μm, range 28–52 μm), all currents exhibited the oscillatory behavior and all completely inactivated during the 200 ms stimulus (mean τ of inactivation, 16.8 ± 2.4 ms). Figure 1B shows a scatter plot of the largest recorded current density versus cell body size for every neuron tested in a series of 133, at the standard holding potential of -70 mV. All but two neurons responded with detectable current (98%; the two non-responders had diameters of 29 μm and 37 μm).

### Current-voltage relationship in standard solutions

**Figure 2:**
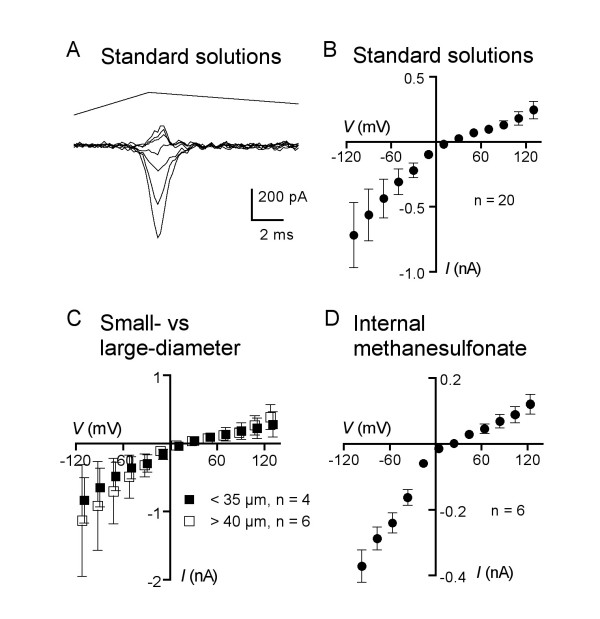
**The mechanically activated current is a non-selective cation current**. (A) Representative traces of mechanotransduction currents evoked by identical mechanical stimuli (probe velocity 4.3 mm/s) from a DRG neuron with a soma diameter of 36 μm. Membrane potential was stepped for 1 s to voltages ranging from -110 to +130 mV in steps of 20 mV, 720 ms prior to mechanical stimulation. Every other trace is omitted for clarity. (B) Mean amplitudes of currents evoked by mechanical stimulation of DRG neurons (n = 20) bathed in the normal external solution and using cesium sulfate/cesium chloride electrode solution. The mean amplitude at -70 mV was -436 ± 145 pA and the mean reversal potential was 25 ± 4 mV. (C) Comparison of *I-V *data for the 4 smallest (<35 μm, closed squares) and 6 largest (>40 μm, open squares) diameter DRG neurons in standard solutions. The two curves are not significantly different from each other, using a 2-way ANOVA test. (D) Mean amplitudes of currents evoked by mechanical stimulation of DRG neurons in the normal external bath solution while using electrodes filled with cesium and the impermeant anion methanesulfonate (n = 6). The mean reversal potential was 23 ± 5 mV.

Our initial report on mechanically induced currents in DRG neurons [6] showed that the MA current is nonselectively carried by sodium and potassium ions. We first determined whether this property was a common characteristic of currents in all DRG neurons. Figure 2A shows a family of currents elicited in a medium-sized DRG neuron (36 μm diameter) at different membrane potentials using standard bath and electrode solutions. Figure 2B shows the mean peak current amplitude versus membrane potential for 20 neurons. The mean reversal potential for these neurons was 25 ± 4 mV, while their mean soma diameter was 39 ± 2 μm (range: 27 to 62 μm). Many characteristics of DRG neurons vary with soma size, reflecting their functional differentiation and especially their stimulus specificity. We asked whether the current-voltage (*I-V*) relation of the MA current varied with DRG soma diameter and therefore compared the mean *I-V *relation of the 4 smallest neurons (<35 μm diam.) to that of the 6 largest ones (>40 μm diam.). Figure 2C shows that, although there was a trend toward smaller outward currents in small-diameter neurons, the difference between the current-voltage relationships of these two groups of neurons was not statistically significant (2-way ANOVA). Notably, there was no significant difference between the mean reversal potentials of the two groups of neurons (small diameter 19 ± 6 mV; large diameter 24 ± 5 mV). Nonselectivity to monovalent ions was thus a consistent property of the whole-cell mechanically activated currents from DRG neurons with disparate sizes.

### Anion permeability

Inward currents at negative intracellular potentials can be carried either by anions (i.e., chloride or sulfate) moving outward across the cell membrane or by inward flowing cations. We therefore tested whether the intracellular (electrode) anions mediate any of the MA current by replacing chloride and sulfate in the electrode solution with the large, relatively channel-impermeant anion methanesulfonate. When cesium methanesulfonate was the primary salt perfused into the intracellular compartment via the recording electrode, mechanically induced whole-cell currents were not appreciably different from those recorded with chloride and sulfate as the major intracellular anions (Fig. 2D, mean amplitude -288 ± 32 pA at -76 mV with methanesulfonate in the electrode (n = 6), versus -436 ± 145 pA at -70 mV using the standard electrode solution (n = 20)). The mean reversal potential for currents recorded with the modified low-chloride electrode solution was 23 ± 5 mV (compare to 25 ± 4 mV with standard electrode solution), which indicates that the MA current is not significantly mediated by electrode anions.

### Monovalent cation selectivity

To characterize the ion selectivity of the mechanosensitive currents we measured the permeability of mechanically stimulated DRG neurons to a variety of different ions relative to that of cesium, the intracellular cation. After establishing the whole cell configuration and adjusting the stimulus protocol to elicit currents of stable amplitude in the standard external solution, bath solutions containing 140 mM of a single cationic species were perfused into the recording chamber. Using the Goldman-Hodgkin-Katz (GHK) equation, the permeability of each ion relative to that of cesium was calculated from the observed reversal potential. We first measured the relative permeability of monovalent cations.

**Figure 3:**
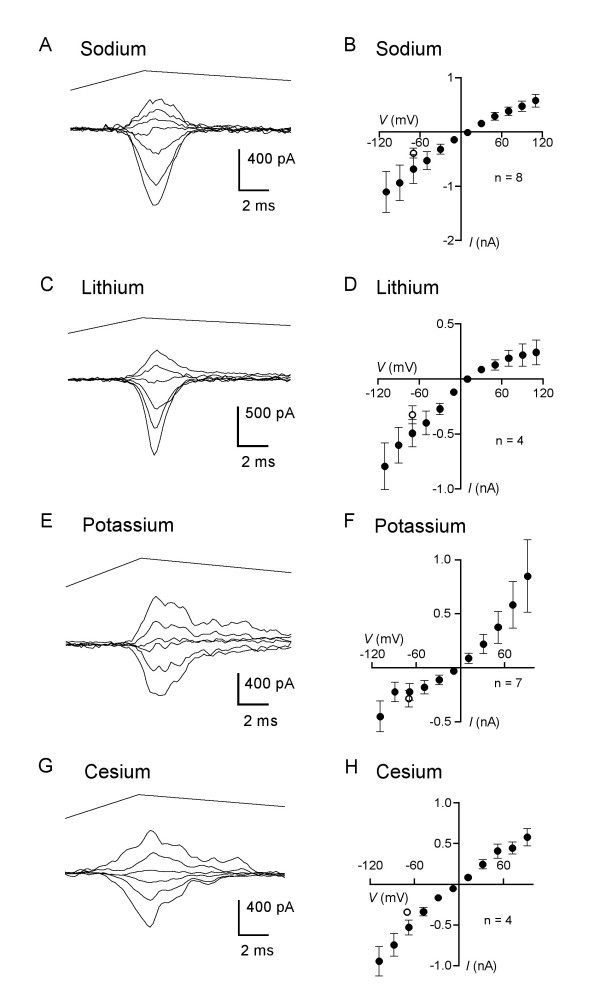
**The mechanotransduction current can be carried by monovalent ions**. (A) Mechanically activated current traces recorded from a DRG neuronal soma with a diameter of 38 μm when external solution consisted of 140 mM NaCl (with HEPES, glucose, and sucrose). Traces shown are for membrane voltages clamped at levels from -110 to +130 mV in increments of 40 mV. Probe velocity was 3.5 mm/s. (B) Mean peak current-voltage relation in sodium chloride bath solution (closed circles). The mean amplitude of peak current seen in standard solutions at -70 mV, in the same cells using the same stimulus parameters, is indicated by the open circle. (C) Current traces recorded from another neuron (41-μm diameter) in LiCl bath solution. Traces shown were recorded at command voltages of -111 to +89 mV in increments of 40 mV. Probe velocity was 3.5 mm/s. (D) Mean current-voltage relation in lithium chloride bath (closed circles). The mean amplitude of current seen in standard solutions at -70 mV, in the same cells using the same stimulus parameters, is indicated by the open circle. (E) Current traces recorded from a 37-μm-diameter DRG neuron when bath solution consisted of 140 mM KCl (with HEPES, glucose, and sucrose) at membrane voltages stepped from -109 to +91 mV in increments of 40 mV. Probe velocity was 4.1 mm/s. (F) Mean current-voltage relation in potassium chloride bath (closed circles). The mean amplitude of current seen in standard solutions at -70 mV, in the same cells using the same stimulus parameters, is indicated by the open circle. (G) Current traces recorded from a neuron (40-μm diameter) in cesium chloride bath with the membrane voltage clamped at -108 to +92 mV in increments of 40 mV. Probe velocity was 3.8 mm/s. (H) Mean current-voltage relation for MA currents in cesium chloride bath and using the potassium-based electrode solution (closed circles). The mean amplitude of current seen with the standard external solution at -70 mV, in the same cells using the same stimulus parameters, is indicated by the open circle.

Figure 3A shows a representative current family recorded with sodium as the only external cation while Figure 3B plots the mean normalized *I-V *relation for 8 neurons recorded under this condition (mean diameter 41 ± 3 μm). The reversal potential for the sodium *I-V *relationship was 7 ± 5 mV (n = 8), from which a Na:Cs permeability ratio of 1.29 is derived (Table 1).

**Table 1:** Reversal potentials and permeability ratios of the mechanotransduction current for different external cations

Ion	n	*V*_Rev _(mV)	P_X_:P_Cs_
Na^+^	8	7.0 ± 4.7	1.29
Li^+^	5	17.0 ± 6.2	1.91
K^+^	7	-6.1 ± 5.2	0.77
Ca^2+^	3	12.6 ± 3.5	1.54
Mg^2+^	6	-7.2 ± 6.1	0.47
Choline	5	-32.9 ± 8.3	0.27
TEA	5	-32.0 ± 7.6	0.28
TRIS	1	-51.4	0.13
NMDG	3	<-111.8	<0.01

We also tested the permeability of lithium, another alkali metal ion. Currents recorded from cells bathed in 140 mM lithium chloride appeared similar to those seen using the sodium bath (Fig. 3A), with a reversal potential of 17 ± 6 mV (n = 5, Fig. 3D). This reversal potential gives a Li:Cs permeability ratio of 1.91 (Table 1).

We then measured the relative ability of the MA current to be carried by potassium ions. Figure 3E shows a representative current family from recordings made with external bath containing 140 mM potassium chloride, while the mean normalized *I-V *relationships for 7 neurons is plotted in Figure 3F. The currents induced in the presence of extracellular potassium typically showed slower inactivation kinetics than those recorded in a sodium-based bath (compare Figs. 3A and 3E). The mean reversal potential for the recordings performed in potassium-based bath was -6 ± 5 mV (n = 7), which indicates a K:Cs permeability ratio of 0.77 (Table 1). Similar results to these were obtained when the bath contained CsCl and the electrode solution was based on K_2_SO_4 _and KCl (Figs. 3G and 3H).

### Divalent cation selectivity

**Figure 4:**
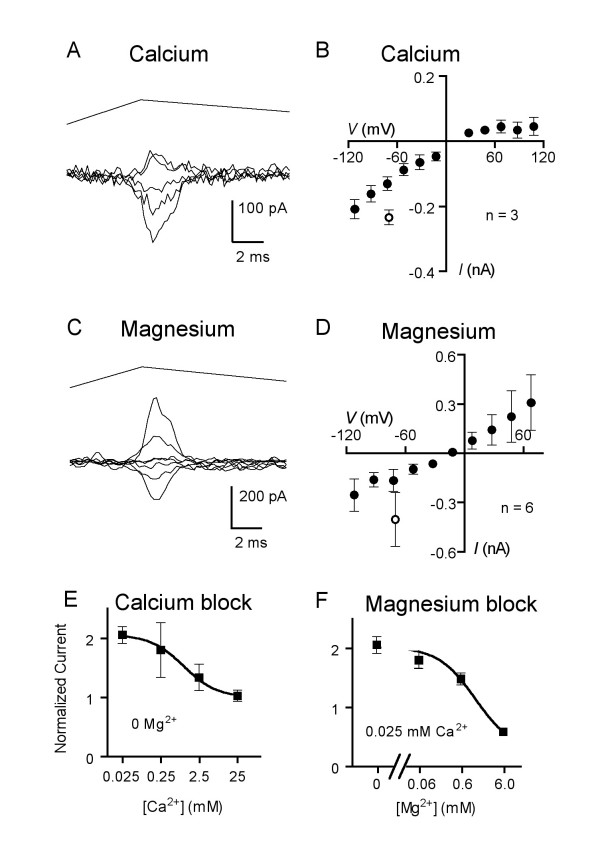
**The mechanotransduction current can be carried and blocked by calcium and magnesium ions**. (A) Mechanically activated current traces recorded from a 40-μm DRG neuronal soma when bath solution consisted of 100 mM CaCl_2 _(with HEPES, glucose, and sucrose) at membrane voltages of -112, -72, -32, +48, and +88 mV. Probe velocity was 3.4 mm/s. (B) Mean current-voltage relation in calcium chloride bath (closed circles). The mean amplitude of current seen in standard solutions at -70 mV, in the same cells using the same stimulus parameters, is indicated by the open circle. (C) Current traces recorded from a neuron (41-μm diameter) when bathed in 100 mM MgCl_2 _at membrane voltages ranging from -112 to +128 mV in steps of 40 mV. Probe velocity was 3.5 mm s^-1^. (D) Mean current-voltage relation for mechanotransduction currents in magnesium chloride bath (closed circles). The mean amplitude of current seen in standard solutions at -70 mV, in the same cells using the same stimulus parameters, is indicated by the open circle. (E) Amplitudes of mechanically activated current responses at -70 mV in otherwise standard external solution containing CaCl_2 _concentrations of 0.025 mM, 0.25 mM, 2.5 mM (standard), and 25 mM and no added MgCl_2_. All current amplitudes were normalized to the size of the current in the same cell (n = 6–8 cells) using the same stimulus parameters in the standard bath solution with 2.5 mM CaCl_2 _and 0.6 mM MgCl_2 _at -70 mV. (F) Amplitudes of responses at -70 mV in bath solutions all containing 0.025 mM CaCl_2 _but with MgCl_2 _concentrations of 0.06 mM, 0.6 mM (standard), and 6 mM. At the left is the value from panel E for 0.025 mM CaCl_2 _and 0 MgCl_2 _for reference. All current amplitudes were normalized to the size of the current in the same cell (n = 3–5 cells) using the same stimulus parameters in the standard bath solution with 2.5 mM CaCl_2 _and 0.6 mM MgCl_2 _at -70 mV.

We next determined the permeability of mechanically activated DRG neurons to divalent cations. When cells were bathed in a test solution of 100 mM CaCl_2_, currents were elicited that reversed at 13 ± 4 mV (n = 3). Using the GHK equation for divalent ions, a permeability ratio of 1.55 relative to cesium was derived (Table 1). Figure 4A shows a current trace family when using calcium as the major external ion, while Figure 4B provides the mean *I-V *relation. When neurons were stimulated in bath containing magnesium ions the elicited currents demonstrated a reversal potential of -7 ± 6 mV (n = 6) and a permeability ratio of 0.46 relative to cesium (Figs. 4C and 4D, Table 1). The calcium currents show an inward rectification (Fig. 4B) while the magnesium currents rectify somewhat outwardly (Fig. 4D).

### Effects of divalent ions on currents carried by monovalents

Mechanotransduction currents described in vertebrate cochlear and vestibular hair cells have demonstrated various degrees of block by calcium ions and other divalent cations [11,12]. In cultured DRG neurons, calcium was shown to block the MA current at millimolar concentrations [7]. In Figure 3 it is evident that inward sodium currents recorded at -70 mV with no external calcium or magnesium are ~60% larger than those from the same neuron in standard bath with 2.5 mM Ca^2+ ^and 0.6 mM Mg^2+^. We thus sought to more completely characterize the effects of divalent ions on the ability of the MA current to be carried by other ions.

For comparison, currents from each neuron were first elicited in bath with standard calcium and magnesium concentrations, after which bath solutions with varying concentrations of one of the divalent cations were substituted. Figure 4E shows the effect of varying the external calcium concentration, in the absence of external magnesium ions but in otherwise standard solutions, where the data for each cell was normalized to current amplitudes observed in the standard, physiologic concentrations of calcium and magnesium (2.5 and 0.6 mM, respectively). All currents elicited in the absence of magnesium, even in the presence of higher than normal calcium (25 mM), were larger than those seen in the standard bath solution with 0.6 mM MgCl_2_. Nevertheless, a dose-dependent partial block of the mechanosensitive current by external calcium is evident. Block of the MA current by calcium was seen in all neurons tested across a wide range of soma diameters (mean: 41 ± 2 μm, range: 34–58 μm, n = 9). We then measured the effect of changing the magnesium concentration of the external solution with calcium at the lowest concentration (0.025 mM), compatible with maintaining the gigaohm seal (Fig. 4F). Again, a clear dose-dependent block of the MA current by magnesium ions was observed to a similar degree as with calcium, and this block was seen in all neurons tested (mean diam.: 46 ± 3 μm, range: 32–57 μm, n = 6).

### Permeability to organic cations

**Figure 5:**
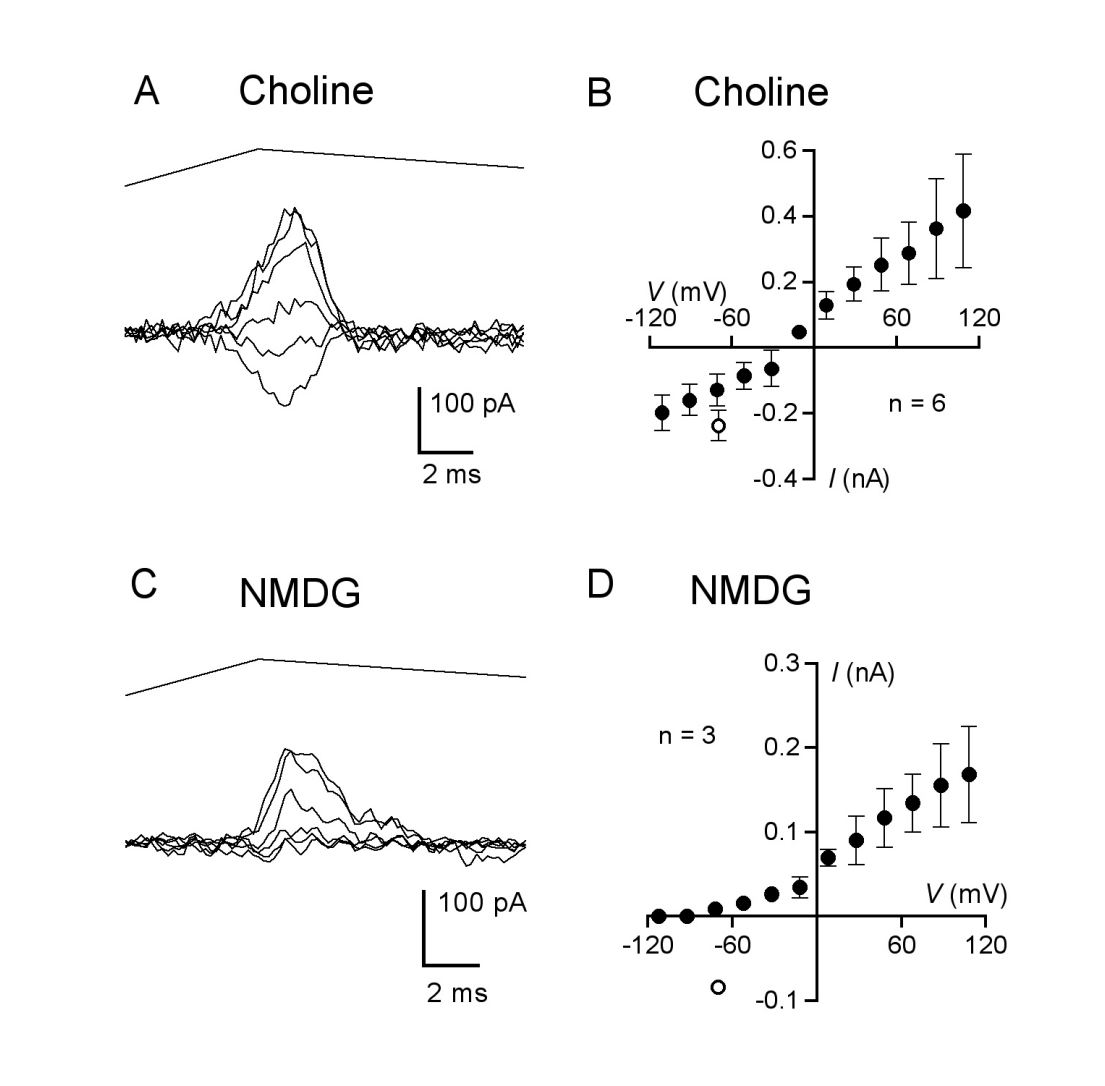
**The mechanotransduction current can be carried by organic ions**. (A) Mechanically activated current traces recorded from a DRG neuron (48-μm diameter) when bath solution consisted of 140 mM choline chloride at membrane voltages stepped from -111 to +89 mV in increments of 40 mV. (B) Mean current-voltage relation in choline chloride bath (closed circles). The mean amplitude of current seen in standard solutions at -70 mV, in the same cells using the same stimulus parameters, is indicated by the open circle. (C) Current traces recorded from a 35-μm neuron when bathed in 140 mM NMDG-Cl with membrane voltage clamped at -112 to +88 mV in increments of 40 mV. Probe velocity was 3.9 mm/s. (D) Mean current-voltage relation for mechanotransduction currents in N-mtheyl-D-glucamine chloride bath (closed circles). The mean amplitude of current seen in standard solutions at -70 mV, in the same cells using the same stimulus parameters, is indicated by the open circle.

To determine the limit of ion selectivity of the channels responsible for the MA current we tested its ability to be carried by organic ions. When cells were bathed in a solution containing 140 mM choline as the major cation, inward currents were elicited (Fig. 5A), demonstrating the permeability of the channels mediating the MA current to this large ion. The currents reversed at mean voltage of -33 ± 8 mV (n = 5) (Fig. 5B), which indicates a choline:Cs permeability ratio of 0.27 (Table 1). Other large organic ions tested include tetraethylammonium (TEA) and tris(hydroxymethyl)aminomethane (Tris), with reversal potentials that indicate permeability ratios of 0.28 and 0.13, respectively (Table 1). In contrast, when the bathing solution contained N-methyl-D-glucamine (NMDG) as the major cation no inward currents could be observed at membrane voltages as negative as -112 mV (Fig. 5C and 5D), so the relative permeability of NMDG ions is less than 1% of that of cesium (Table 1). Thus, large organic ions are able to permeate the channels responsible for mechanically activated DRG currents, indicating a pore size comparable to the muscle endplate nicotinic acetylcholine receptor [13].

## Discussion

We have described the ionic selectivity and block by divalent cations of a mechanically activated whole-cell current [6], in mammalian somatosensory neurons *in vitro*. The MA current was non-selective for cations but was not carried by chloride or sulfate ions. The ability of this current to pass organic ions such as choline and Tris demonstrates that the pore of the underlying channel must be relatively large, while the inability to pass NMDG ions at voltages as negative as -112 mV further confirms that the current is not simply the result of membrane damage [6]. While the current exhibits significant ability to be carried by divalent cations, both calcium and magnesium at physiological external concentrations also cause a partial block of the primary sodium conductance of the MA current. Our findings largely concur with the reported cation nonselectivity and permeability to divalents of stretch activated single channels in DRG neurons [3], which may thus underlie the macroscopic current observed here. These results represent one the few cellular studies to characterize the poorly understood process of the transduction of direct mechanical stimuli by somatosensory neurons [6,7,9] and is the first detailed description of the ion selectivity of whole-cell mechanotransduction currents.

The mechanically induced current described here was recorded using the cultured cell soma with minimal neurite outgrowth, an experimental model for the peripheral terminals of somatosensory neurons [14-20]. While it should be acknowledged that this *in vitro *system is significantly different from *in vivo *nerve endings, especially regarding the geometry and immediate environment of the transduction site, it has been successfully used to replicate the sensitization by hyperalgesic agents [17], as well as the transduction of chemical [14-16] and thermal [18-20] stimuli, that normally occur in the peripheral terminals of sensory neurons. It is possible that the axotomy of DRG cell bodies causes the cultured soma to express the signaling apparatus normally expressed in peripheral terminals [21].

The expression of mechanosensitive currents in 98% of DRG neurons tested in this report contrasts with another report in mouse DRG neurons showing 70% of large diameter and 51% of small-medium diameter neurons responding to mechanical stimulation [8], and our previous paper that reported such currents in 70% and 35% of large- and small diameter adult rat DRG neurons, respectively [6], but is similar to results with positive pressure applied through the recording pipette where ~90% of neonatal rat DRG neurons 25 μm or greater in diameter responded [10]. The high rate of mechanosensitivity seen in our *in vitro *experiments also contrasts with observations of ~25% of primary afferent fibers being mechanically insensitive *in vivo *[22]. The difference in the *in vitro *results may be caused by the use of different stimulus protocols, experimental animals, or cell culture methods. In addition, there may be a selection bias in the current study toward larger, healthier neurons with a more rounded vertical profile since it is easier to maintain recordings when such neurons are mechanically stimulated. The *in vivo *mechanically insensitive afferents [22] were primarily C-fiber nociceptors. It is possible that some high-threshold fibers may not respond to mechanical stimulation of the skin while their corresponding small-diameter cell bodies are mechanically responsive to direct stimulation *in vitro*. More importantly, mechanically insensitive fibers develop mechanical sensitivity following exposure to an inflammatory soup [23,24], and may also do so after injury such as upon dissection of the dorsal root ganglion for culture.

Of note, only one stimulation protocol was employed in this study. Probes moving with different velocities or temporal patterns (e.g., vibration) may activate additional channels and thus evoke currents with different properties. For example, while the currents recorded here exhibited only a transient, rapidly inactivating component, we and others have recorded currents with sustained or slowly inactivating components using a similar probe applied with a lower velocity and at a steeper angle of approach or using a hydraulic jet that causes a presumably dynamic and sustained displacement of the plasma membrane [6,7]. In addition, the DRG neurons used in this study were cultured in the presence of 50 ng/ml nerve growth factor (NGF). A recent report has shown that culturing in growth media containing 100 ng/ml NGF significantly increases the expression of MA currents *in vitro *[9]. While all of the previously referenced studies of MA currents in DRG neurons also used NGF at similar concentrations, the combination here of optimal growth conditions, fast stimulation protocol, and potential selection bias may have allowed the observation of MA currents in nearly all neurons tested.

There were no appreciable differences in our measurements of ion selectivity of MA currents between neurons of different sizes. Since the conduction velocities, and hence presumed receptive functions, of DRG neurons are roughly segregated by cell soma diameter [25,26], our results did not distinguish any differences in the mechanotransduction currents across sensory neuron functional types (i.e., between Aα-, Aβ-, Aδ-, and C-fibers). Drew *et al*. found differences in sensitivity to mechanical stimulation and to block by calcium ions between capsaicin-sensitive and -insensitive small diameter DRG neurons [7]. Our results show a generalized sensitivity to mechanical stimulation and block by divalent cations across all soma sizes. The variation in sensitivity and stimulus specificity seen in somatosensory neurons *in vivo *may be explained, at least in part, by the effects of support cells (e.g., Merkel discs), specialized terminal structures (e.g., Pacinian corpuscles) or other details of the immediate environment of the transduction sites.

The ability of mechanically activated currents in DRG neurons to carry organic ions as large as choline is revealing and may be an additional marker for identification of the responsible channel. The evidence presented here of a large mechanotransduction channel pore is consistent with reports of the ability of the styryl dye FM1-43 to permeate the mechanotransduction channels of hair cells [27], and, possibly, of somatosensory neurons [28]. This property may also be exploited for further characterization and identification of the mechanotransducer. Interestingly, FM1-43 also permeates TRPV1 channels [28]. Since FM1-43 was seen to all sizes of DRG neuron, which would include ones not expressing TRPV1, there may be other related channels that allow entry of the dye into the somatosensory neurons.

## Conclusion

Taken together, these data show that the channels that carry somatosensory mechanosensitive currents are non-selective to cations, pass organic ions, and have a significant calcium conductance. These results allow a comparison of these MA currents to those carried by channels that are suspected of being mammalian somatosensory mechanotransducers. The two leading candidate channel families are the DEG/ENaC (degenerin/epithelial sodium channel) family and the TRP (transient receptor potential) family [29]. The cation non-selectivity makes it unlikely that a member of the sodium-selective DEG/ENaC family primarily mediates the MA current observed here. The fact that the MA current and the vanilloid receptor subtypes of TRP channel (TRPVs) are both blocked by ruthenium red [8,20], as well as that FM1-43 permeates TRPV1 and can selectively enter mechanosensory neurons [28], are suggestions of a possible role for a TRP member in mechanotransduction. Other members of this channel family are strongly implicated in mechanotransduction in *Drosophila *sensory bristles and in hearing in flies, zebrafish, and mammals [30-34]. While DEG/ENaC family members are clearly necessary for mechanotransduction in *C. elegans *[35], at least two mammalian members that are present in DRG neurons do not appear to participate in mechanotransduction *in vitro *[8]. This channel family may play a role in other possible forms of mechanotransduction in sensory neurons. Since touch and mechanosensation in general are so critical to survival, it is likely that they are mediated by redundant mechanisms, which may include different combinations of heterologous subunits [33].

The nature of the channel(s) that mediate mechanical transduction in primary afferent neurons is obscure. Unlike with other sensory transduction processes, there has been a lack of an easily observed *in vitro *or *in vivo *model in which to collect the data at the cellular level that is required for the characterization of such a channel. Taken together with the few earlier reports of electrophysiologically observed mechanical transduction in cultured DRG neurons [6-9], this study allows the formation of new hypotheses regarding the nature and identity of mammalian mechanical transduction channels.

## Methods

### DRG cell culture

All chemicals for cell culture and electrophysiology were obtained from Sigma (St. Louis, MO, USA) unless otherwise stated. Lumbar dorsal root ganglia were dissected from adult male Sprague-Dawley rats (Charles River Laboratories, MA, USA) anaesthetized with 75 mg/kg sodium pentobarbital (Abbott Laboratories, IL, USA). After removal of the ganglia, rats were killed by overdose of anesthetic and cervical dislocation. Care and use of rats was in accordance with IASP, NIH, and UCSF guidelines, and experiments were approved by the UCSF Committee on Animal Research. Ganglia were desheathed and placed in growth medium – minimal essential medium supplemented with vitamins, antibiotics, 10% fetal bovine serum, and 50 ng/ml nerve growth factor (Roche Applied Science, IN, USA) – with 0.125% collagenase at 37°C for 90 min, similar to previously described methods [36]. Ganglia were transferred to Ca^2+^- and Mg^2+^-free Hank's basic salt solution containing 0.125% trypsin (Worthington, NJ, USA) for 10 minutes at 37°C after which trypsinization was quenched with an equal volume of growth medium with 2.5 mg/ml MgSO_4_. The ganglia were triturated using fire-polished Pasteur pipettes with progressively smaller bores and the dissociated cells were plated onto coverslips coated with poly-ornithine and laminin (Invitrogen, CA, USA). Cells were maintained in growth medium at 37°C with humidified air containing 3% CO_2 _and used for experiments within 24 hours of plating, prior to significant neurite outgrowth.

### Electrophysiology

Patch-clamp recordings were performed using fire-polished borosilicate electrodes with resistances of 1.5–4 MΩ.  Electrodes were filled with a solution containing (mM): 55 Cs_2_SO_4_, 30 CsCl, 2 MgCl_2_, 10 HEPES (pH 7.2-CsOH, 325 mOsm, final Cs concentration 143 mM) while the standard external solution consisted of (mM): 130 NaCl, 3 KCl, 0.6 MgCl_2_, 2.5 CaCl_2_, 10 HEPES, 10 glucose (pH 7.4, 335 mOsm). Some earlier experiments (used for data in Fig. 1 only), as well as those in external cesium (Figs. 3G and 3H), were performed using a potassium-based electrode solution containing (mM): 55 K_2_SO_4_, 30 KCl, 2 MgCl_2_, 10 HEPES (pH 7.2-KOH, 325 mOsm). Cation substitution experiments were performed with single-cation solutions consisting of, for monovalent cations, (mM): 140 XCl, 10 HEPES, 10 glucose (where X = cation of interest, pH 7.4, 335 mOsm). Solutions using divalent cations consisted of (mM): 110 XCl_2_, 10 HEPES, 10 glucose (pH 7.4-, 335 mOsm). Anion substitution experiments were performed with the standard bath solution and an electrode solution consisting of (mM): 130 methanesulfonic acid, 2 MgCl_2_, 10 HEPES (pH 7.2-CsOH, 325 mOsm). The osmolarity of all solutions was adjusted with sucrose and the pH was adjusted with Trizma^® ^base unless otherwise noted. Signals were amplified by an Axopatch 200B (Axon Instruments, CA, USA) and recorded and analyzed using the pClamp suite of programs (Axon Instruments). Liquid junction potentials (8.8–15.8 mV) were calculated for each combination of bath and electrode solutions using Clampex, and command voltages were corrected accordingly *a posteriori*.

### Mechanical stimulation

Dorsal root ganglion neurons were mechanically stimulated as previously described [6]. The tip of a borosilicate glass micropipette, identical to those used for recording electrodes, was melted to make it blunt and to seal the opening, resulting in a tip diameter of ~3–6 μm. The micropipette was mounted on a piezoelectric stage so that it moved toward the cell at a trajectory that was approximately 20° from horizontal. The voltage output to drive the stage was controlled by a protocol in the pClamp program. The original position and distance traveled by (and hence velocity of) the probe was adjusted to produce large currents without disrupting the whole-cell recording. The probe was held a horizontal distance of 12 μm from the lateral edge of the cell as seen through the microscope and, when activated, contacted the cell at a point 7–13 μm in from the edge. The probe was driven a total of 30 to 45 μm over 10 ms (3–4.5 mm s^-1^), therefore it traveled 5–20 μm further after contacting the cell membrane. The low angle of approach allowed the distance that the probe traveled after contacting the cells to not be as limited by the relatively small vertical dimension of the cell. The probe was typically withdrawn immediately over 40 ms, but sometimes was held at the final position for 200 ms and then withdrawn (Fig. 1).

### Determination of current-voltage relations and permeability ratios

The membrane potential of a neuron was stepped for 1 s to a series of command voltages between -100 and +120 mV in 20 mV increments. During each step a standard 50-ms mechanical stimulus was delivered. The mechanical stimuli were delivered 700 ms after the voltage was stepped to allow the majority of voltage-activated membrane currents to inactivate. For determination of current-voltage (*I-V*) relationships we used a cesium-based electrode solution to reduce the outward delayed rectifier currents that could obscure small mechanically induced currents at positive potentials. For a crude comparison of the amplitude of inward currents elicited in the various single cation external solutions to the amplitude of those seen in standard sodium-based bath solution, the mean amplitude of 3–5 current responses seen in each cell in standard external solution (before switching to the test solution), at the holding potential of -70 mV, is indicated by an open circle in each *I*-*V *plot. Reversal potentials for each cell in each solution were determined by interpolation on fitted curves of the respective *I-V *data. Data are given as means ± S.E.M.

The ratio of permeabilities, P_X_/P_Cs_, was determined for each test cation X from the mean reversal potential of the mechanically activated whole-cell current when that cation was the major external ion. The Goldman-Hodgkin-Katz (GHK) equation [37], simplified for a single permeant ion on each side of the membrane, was employed:

Erev=RTzFln⁡PX[X]oPCs[Cs]i
 MathType@MTEF@5@5@+=feaafiart1ev1aaatCvAUfKttLearuWrP9MDH5MBPbIqV92AaeXatLxBI9gBaebbnrfifHhDYfgasaacH8akY=wiFfYdH8Gipec8Eeeu0xXdbba9frFj0=OqFfea0dXdd9vqai=hGuQ8kuc9pgc9s8qqaq=dirpe0xb9q8qiLsFr0=vr0=vr0dc8meaabaqaciaacaGaaeqabaqabeGadaaakeaacqWGfbqrdaWgaaWcbaGaeeOCaiNaeeyzauMaeeODayhabeaakiabg2da9maalaaabaGaemOuaiLaemivaqfabaGaemOEaONaemOrayeaaiGbcYgaSjabc6gaUnaalaaabaGaemiuaa1aaSbaaSqaaiabbIfaybqabaGccqGGBbWwcqqGybawcqGGDbqxdaWgaaWcbaGaee4Ba8gabeaaaOqaaiabdcfaqnaaBaaaleaacqqGdbWqcqqGZbWCaeqaaOGaei4waSLaee4qamKaee4CamNaeiyxa01aaSbaaSqaaiabbMgaPbqabaaaaaaa@4D40@

where *RT/zF *has the value of 25.5 mV at 23°C. For the divalent cations the appropriately modified equation was used:

Erev=RTFln⁡{4PCa[Ca]oPCs[Cs]i+14−12}.
 MathType@MTEF@5@5@+=feaafiart1ev1aaatCvAUfKttLearuWrP9MDH5MBPbIqV92AaeXatLxBI9gBaebbnrfifHhDYfgasaacH8akY=wiFfYdH8Gipec8Eeeu0xXdbba9frFj0=OqFfea0dXdd9vqai=hGuQ8kuc9pgc9s8qqaq=dirpe0xb9q8qiLsFr0=vr0=vr0dc8meaabaqaciaacaGaaeqabaqabeGadaaakeaacqWGfbqrdaWgaaWcbaGaeeOCaiNaeeyzauMaeeODayhabeaakiabg2da9maalaaabaGaemOuaiLaemivaqfabaGaemOrayeaaiGbcYgaSjabc6gaUnaacmqabaWaaOaaaeaadaWcaaqaaiabisda0iabdcfaqnaaBaaaleaacqqGdbWqcqqGHbqyaeqaaOGaei4waSLaee4qamKaeeyyaeMaeiyxa01aaSbaaSqaaiabb+gaVbqabaaakeaacqWGqbaudaWgaaWcbaGaee4qamKaee4CamhabeaakiabcUfaBjabboeadjabbohaZjabc2faDnaaBaaaleaacqqGPbqAaeqaaaaakiabgUcaRmaalaaabaGaeGymaedabaGaeGinaqdaaaWcbeaakiabgkHiTmaalaaabaGaeGymaedabaGaeGOmaidaaaGaay5Eaiaaw2haaiabc6caUaaa@57F3@

## Competing interests

The author(s) declare that they have no competing interests.

## Authors' contributions

GM designed and carried out all of the experiments and wrote the manuscript. JL provided material, conceptual, intellectual, and editorial support. Both authors read and approved the final manuscript.
